# Neuroimaging of tissue microstructure as a marker of neurodegeneration in the AT(N) framework: defining abnormal neurodegeneration and improving prediction of clinical status

**DOI:** 10.1186/s13195-023-01281-y

**Published:** 2023-10-17

**Authors:** Rigina L. Gallagher, Rebecca Langhough Koscik, Jason F. Moody, Nicholas M. Vogt, Nagesh Adluru, Steven R. Kecskemeti, Carol A. Van Hulle, Nathaniel A. Chin, Sanjay Asthana, Gwendlyn Kollmorgen, Ivonne Suridjan, Cynthia M. Carlsson, Sterling C. Johnson, Douglas C. Dean, Henrik Zetterberg, Kaj Blennow, Andrew L. Alexander, Barbara B. Bendlin

**Affiliations:** 1 School of Medicine and Public Health, University of Wisconsin, Madison, WI USA; 2 Medical Scientist Training Program, University of Wisconsin-Madison, Madison, WI USA; 3 Neuroscience Training Program, University of Wisconsin-Madison, Madison, WI USA; 4 Wisconsin Alzheimer’s Disease Research Center, Madison, WI USA; 5 Wisconsin Alzheimer’s Institute, Madison, WI USA; 6 Waisman Research Center, Madison, WI USA; 7 Veterans Administration, Madison, WI USA; 8 Roche Diagnostics GmbH, Penzberg, Germany; 9 Roche Diagnostics International Ltd, Rotkreuz, Switzerland; 10 Department of Psychiatry and Neurochemistry, Institute of Neuroscience and Physiology, The Sahlgrenska Academy at the University of Gothenburg, Mölndal, Sweden; 11 Clinical Neurochemistry Laboratory, Sahlgrenska University Hospital, Mölndal, Sweden; 12 Department of Neurodegenerative Disease, UCL Institute of Neurology, Queen Square, London, UK; 13 UK Dementia Research Institute at UCL, London, UK

**Keywords:** Neuroimaging, Diffusion-weighted imaging, CSF biomarkers, Alzheimer’s disease, Neurodegeneration

## Abstract

**Background:**

Alzheimer’s disease involves accumulating amyloid (A) and tau (T) pathology, and progressive neurodegeneration (N), leading to the development of the AD clinical syndrome. While several markers of N have been proposed, efforts to define normal vs. abnormal neurodegeneration based on neuroimaging have been limited. Sensitive markers that may account for or predict cognitive dysfunction for individuals in early disease stages are critical.

**Methods:**

Participants (*n* = 296) defined on A and T status and spanning the AD-clinical continuum underwent multi-shell diffusion-weighted magnetic resonance imaging to generate Neurite Orientation Dispersion and Density Imaging (NODDI) metrics, which were tested as markers of N. To better define N, we developed age- and sex-adjusted robust *z*-score values to quantify normal and AD-associated (abnormal) neurodegeneration in both cortical gray matter and subcortical white matter regions of interest. We used general logistic regression with receiver operating characteristic (ROC) and area under the curve (AUC) analysis to test whether NODDI metrics improved diagnostic accuracy compared to models that only relied on cerebrospinal fluid (CSF) A and T status (alone and in combination).

**Results:**

Using internal robust norms, we found that NODDI metrics correlate with worsening cognitive status and that NODDI captures early, AD neurodegenerative pathology in the gray matter of cognitively unimpaired, but A/T biomarker-positive, individuals. NODDI metrics utilized together with A and T status improved diagnostic prediction accuracy of AD clinical status, compared with models using CSF A and T status alone.

**Conclusion:**

Using a robust norms approach, we show that abnormal AD-related neurodegeneration can be detected among cognitively unimpaired individuals. Metrics derived from diffusion-weighted imaging are potential sensitive markers of N and could be considered for trial enrichment and as outcomes in clinical trials. However, given the small sample sizes, the exploratory nature of the work must be acknowledged.

**Supplementary Information:**

The online version contains supplementary material available at 10.1186/s13195-023-01281-y.

## Introduction

Amyloid plaques and neurofibrillary tangles (NFTs) are defining features of Alzheimer’s disease (AD) [1]. As underscored by the AT(N) framework, determining positivity for these features either by way of neuroimaging or deriving cutoffs based on cerebrospinal fluid (CSF) levels of Aβ42 and p-Tau can better define the disease, prior to development of the clinical syndrome of AD [2–5]. Cerebrospinal fluid (CSF) levels of Aβ42 and p-Tau have become widely adopted in AD research as sensitive markers for preclinical AD, where pathology pre-dates clinical manifestations of AD [2–4, 6]. Identifying disease markers is important for early identification as well as for enriching therapeutic intervention trials. Biomarkers of AD can also provide quantitative benchmarks that may evaluate the efficacy of disease-modifying drugs [4]. CSF has proven to be highly useful and provides several biomarkers from a single sample; however, CSF biomarkers lack the regional specificity of neuroimaging techniques, potentially missing key AD-defining patterns of pathology. Neurodegeneration (N) is a critical facet of the AD process, since it likely mediates the relationship between amyloid plaques and tangles and clinical cognitive decline [7]. However, the development and utility of neuroimaging-based markers of N has been limited, given that conventional magnetic resonance imaging (MRI) based measures of volume primarily capture the gross atrophy that is typical of later stage disease, and disease-specific neurodegeneration may be difficult to parse from age-related changes [8, 9].

Recently, several quantitative microstructural MRI techniques, including multi-shell diffusion-weighted MRI (dMRI), T2 relaxometry, quantitative magnetization transfer (qMTR), and cortical mean diffusivity (cMD) have emerged as promising for detecting microstructural alterations that precede the gross anatomic changes observed with conventional T1-weighted imaging in both cortical gray matter and subcortical white matter tracts [10–15]. By definition, quantitative MRI is more sensitive to tissue type than conventional MRI [13]. T2 relaxometry and DTI indices examined in medial temporal lobe regions have improved the sensitivity and specificity of detecting amnestic MCI and AD compared to conventional FA and MD [13]. Mole et al. [14] have also quantified the multiple interaction effects of *APOE-ε4*, family history of dementia, and obesity on white matter microstructure over time using quantitative magnetization transfer (qMTR). In recent years, multi-shell acquisition techniques coupled with novel modelling approaches have enhanced capabilities for deriving biologically plausible estimates of brain microstructural architecture in vivo [9, 16–18]. Studies suggest that cortical mean diffusivity (cMD) can detect preclinical changes in gray matter architecture that correlate with biomarker status [15]. In addition, Neurite Orientation Dispersion and Density Imaging (NODDI), a type of multi-shell acquisition DWI, has gained increased use for identifying microstructural alterations in neuropsychiatric and neurodegenerative diseases including, schizophrenia, bipolar disorder, Parkinson’s disease, and AD [19–24]. Previous work from our group has shown that NODDI metrics are especially sensitive to cortical microstructural alterations across the AD continuum, outperforming cortical thickness at predicting both MCI and AD [25] as well as exhibiting higher sensitivities to CSF markers of AD pathology [25].

NODDI—which has shown promise for capturing subtle neurodegeneration—applies a three-compartment tissue model [18]. By assuming three separate environments for water diffusion, NODDI accounts for both intracellular and extracellular water diffusion, allowing for a better interpretation of the neurodegenerative changes that occur in aging and AD [18]. The Neurite Density Index (NDI) estimates the density of axons and dendrites, or “neurites,” per voxel, and the Orientation Density Index (ODI) estimates the degree of neurite dispersion. ODI is impacted by loss of neurites, particularly when measured in gray matter. NODDI has gained increased use for identifying microstructural alterations in neuropsychiatric and neurodegenerative diseases including, schizophrenia, bipolar disorder, Parkinson’s disease, and AD [19–24].

To better define abnormal neurodegeneration (N) within the AT(N) framework, the current study leveraged multi-shell dMRI and NODDI modeling of data collected among participants who spanned the clinical and biological spectrum of AD. We applied a robust norms statistical approach (previously utilized for norming neuropsychological tests), to establish normative ranges and cut-offs for neurodegeneration (N) [26–28] in gray and white matter regions of interest. Using robust norms analysis of NODDI-ODI and NDI measures, we aimed to better identify preclinical neurodegeneration within AD-affected cortical and subcortical regions utilizing AT(N) biomarker criteria.

We hypothesized that the robust values of ODI and NDI in AD-affected regions would have an inverse relationship with participant clinical severity and AT biomarker status indicative of neurodegenerative loss of axons and dendrites with worsening AT(N) disease. Currently, the AT(N) framework prioritizes biomarkers of A + and T + to categorize AD staging. However, since neurodegeneration likely mediates the relationship between A/T pathology and clinical cognitive decline in AD [6], we hypothesized that models that utilized regional NODDI metrics would better predict clinical diagnosis status than models that relied on CSF A/T status alone. Here, we show that microstructural alterations in AD-associated regions could be separated into normal vs. abnormal neurodegeneration (N) in preclinical and clinical AD stages, as well as inform upon the clinical predictive utility of relying on neuroimaging markers of neurodegeneration, such as NODDI, in addition to CSF biomarkers. While our work is not without limitation, and further investigation is needed to fully define N within the AT(N) framework, we demonstrate that microstructural diffusion-weighted imaging, particularly NODDI, has potential as a neuroimaging biomarker for the detection of preclinical neurodegenerative (N) changes in AD.

## Methods

### Participant selection

Data from 296 participants (Table 1) enrolled in the Wisconsin Registry for Alzheimer’s Prevention study (WRAP, *n* = 90), or the Wisconsin Alzheimer’s Disease Research Center (ADRC, *n* = 206) were included in analyses [21, 29]. Selection criteria included having undergone multi-shell diffusion-weighted MRI to determine NODDI metrics, successful lumbar puncture (LP) to measure Aβ42, Aβ42/40, and p-Tau via the Roche NeuroToolKit (NTK) platform, and a clinical consensus diagnosis of either cognitively unimpaired (CU, *n* = 285), mild cognitive impairment likely due to AD (MCI, *n* = 6), or dementia, due to probable AD (AD, *n* = 5). To ensure the analysis was focused on individuals on the AD continuum, selected participants were required to have either (1) a clinical diagnosis of either MCI or AD with a positive amyloid CSF status (A + /T − , A + /T +), or (2) a diagnosis of CU with either A − /T − , A + /T − or A + /T + CSF status. For each participant, the LP sample and clinical diagnosis closest in time to the latest NODDI scan was included in analysis. In accordance with NIA-AA guidelines, those classified as MCI likely due to AD, underwent clinical evaluation, brain imaging, neuropsychological testing, and for simplicity are referred to as MCI.

**Table 1:** Participant demographics

Participant characteristic	Cognitively unimpaired (CU)	Mild cognitive impairment (MCI-AD)	Alzheimer’s disease dementia (dementia-AD)	Statistical method	***p***-value
***N*** = 296	*N* = 285	*N* = 6	*N* = 5		
CSF Biomarker Status (A − T − /A + T − /A + T +)	231/26/28 (81.0%|9.1%|9.8%)	0/1/5 (0%|16.7%|83.3%)	0/1/4 (0%|20.0%|80.0%)	Fisher	1.07 × 10^–8^
*APOE* ε4 genotype (% positive)	103(36%) NA = 10	3 (50%)	4(80%)	Fisher	0.026
Sex (% female)	186 (65%)	5 (83%)	2 (40%)	Fisher	0.41
Age (years) Mean, SD	65.10 ± 7.81	71.77 ± 9.73	71.36 ± 2.52	ANOVA	0.0121

Two hundred ninety-six participants from the Wisconsin Alzheimer’s Disease Research Center (ADRC) and the Wisconsin Registry for Alzheimer’s Prevention (WRAP) studies at UW-Madison were included in the study. Participants underwent lumbar puncture (LP) for cerebrospinal fluid (CSF) collection and assays for Aβ42/40 and p-Tau, clinical diagnosis, MRI studies, and NODDI modeling. Exclusion criteria included a diagnosis other than AD-Dementia, MCI-AD, or CU. Participants were selected if they had a clinical diagnosis of CU with A − /T − , A + /T − , or A + /T + CSF or a diagnosis of MCI or AD and A + /T − or A + /T + CSF. CSF was analyzed using the Roche NeuroToolKit (NTK) assay. CSF cutoffs were determined in-house using previously published receiver operator curve (ROC) methods

### Clinical diagnosis categories

Participants underwent comprehensive cognitive assessments. Diagnosis of cognitively unimpaired (CU, *n* = 285), mild cognitive impairment (MCI, *n* = 6) likely due to AD, or dementia due to probable AD (AD, *n* = 5) was determined using clinical and cognitive information in accordance with the 2011 National Institute on Aging-Alzheimer’s Association (NIA-AA) workgroup diagnostic criteria. MCI and AD groups were combined for the primary analysis (MCI/AD) due to small sample size, and statistical approaches for small and unequal sample sizes were employed.

### CSF AD biomarker acquisition and A/T status analysis

CSF samples were collected via lumbar puncture (LP) after a minimum 8-h fast, centrifuged, aliquoted, and stored at − 80 °C [30]. CSF samples were assayed at the Clinical Neurochemistry Laboratory, University of Gothenburg. Measurements included the following immunoassays performed on cobas e analyzers: Elecsys® β-Amyloid (1–42) CSF, Elecsys Phospho-Tau (181P) CSF and the β-Amyloid (1–40) robust prototype assay as part of the Roche NeuroToolKit (NTK) research platform.

Cutoff values for amyloid and p-Tau positivity were applied according to Van Hulle et al. (2021) [29, 30] as follows: a CSF Aβ42/Aβ40 ratio < 0.046 defined amyloid positivity (A +), and a CSF p-Tau value ≥ 24.8 defined tau positivity (T +). Participants were then placed into one of three A/T status groups: A − /T − (Amyloid-/Tau-), A + /T − (Amyloid + /Tau −), and A + /T + (Amyloid + /Tau +). A − /T + participants were not used as they were outside the AD A/T-criteria focus of this study [2]. Among the *N* = 285 CU participants, 54 were defined as having biological AD based on their CSF A/T biomarker status (A + /T −  = 16, and A + /T +  = 28).

### Image acquisition and processing

MRI data were acquired on two General Electric 3 T MR750 scanners with 32-channel head coils located at the Waisman Center (*n* = 90) or the Wisconsin Institute for Medical Research (WIMR) (*n* = 206) at UW-Health, Madison WI. Diffusion-weighted images were acquired using a multi-shell spin-echo echo-planar imaging pulse sequence (6 × b = 0 s mm^2^, 9 × b = 500 s/mm^−2^, 18 × b = 800 s mm^−2^, and 36 × b = 2000s mm^−2^; TR/TE = 8575/76.8 ms; 2 × 2 × 2 mm^3^ isotropic voxel resolution; 128 × 128 acquisition matrix). T1-weighted structural images were acquired using a 3D inversion recovery prepared fast spoiled gradient-echo FSPGR-BRAVO sequence (TI = 450 ms; TR/TE = 8.1/3.2 ms; flip angle = 12; 1 × 1 × 1mm^3^).

Similar to Vogt et al. (2020), diffusion-weighted images were denoised [21, 31] and corrected for Gibb’s ringing [32, 33] using MRtrix3 [34], and then motion-corrected and eddy current distortion corrected using the eddy tool [31] in FSL (v5.0.11) [33]. Diffusion tensor fitting was performed using Diffusion Imaging in Python (DIPY) [32] to generate fractional anisotropy (FA) maps. NDI, ODI, and Viso parameter maps were generated by fitting the NODDI model in Python using Accelerated Microstructure Imaging via Convex Optimization (AMICO), which improves processing speed by approximating the NODDI model as a linear system [35]. Given that diffusion parameters likely vary between tissue types [18, 36] two sets of NODDI images were generated: one using the original intrinsic parallel diffusivity of 1.7 μm^−2^ ms^−1^ which were used for white matter NODDI value extraction, and second set using a gray matter optimized intracellular intrinsic parallel diffusivity of 1.1 μm^−2^ ms^−1^ [36] which were used for gray matter NODDI value extraction.

### Region of interest (ROI) selection and analysis

**Fig. 1:**
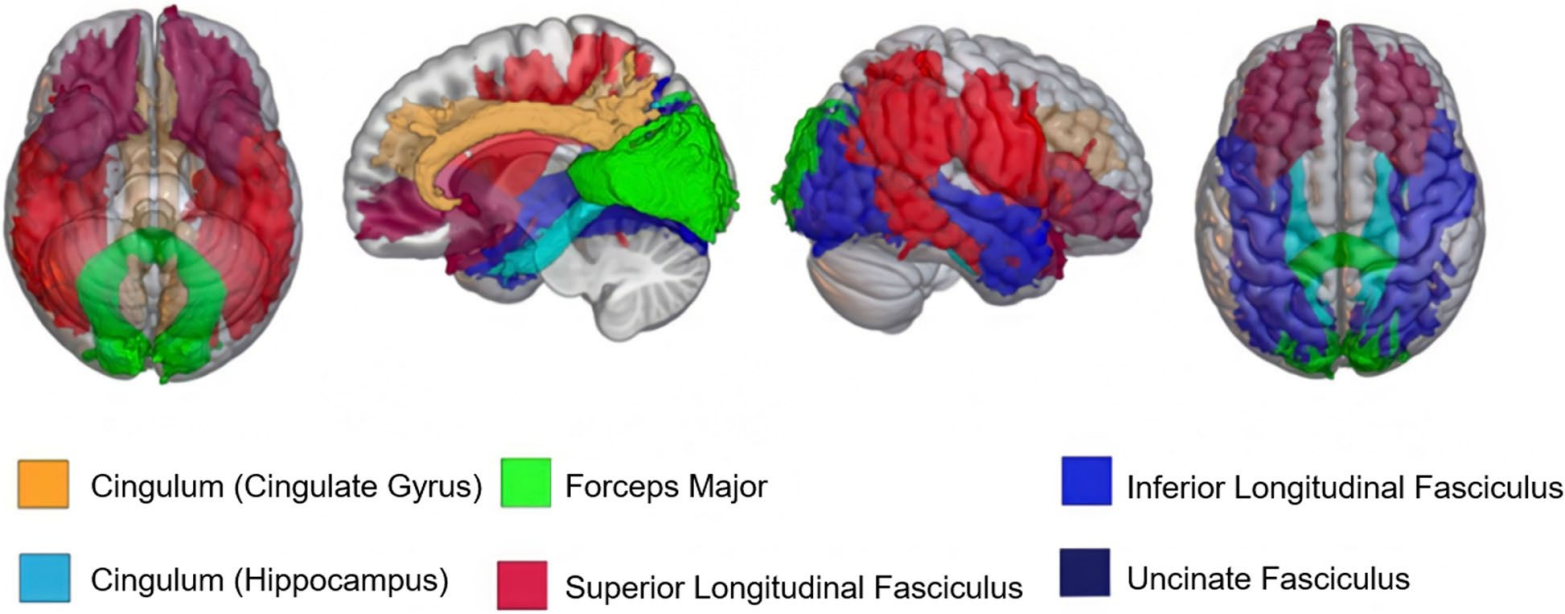
Representation of white matter regions of interest (ROIs). Left to right: inferior axial, midsagittal, parasagittal, and superior axial views of final white matter ROIs. NDI and ODI were extracted from six bilateral white matter ROIs using the JHU-Tractography Atlas, and then averaged bilaterally before inclusion into logistic regression models. The ROI figure was constructed in MRIcroGL using the JHU-Tractography Atlas. Regions correspond to figure legend and are as follows: green = forceps major, light blue = cingulum (hippocampus), dark blue = inferior longitudinal fasciculus, orange = cingulum (cingulate gyrus), red = superior longitudinal fasciculus, purple-black = uncinate fasciculus

**Fig. 2:**
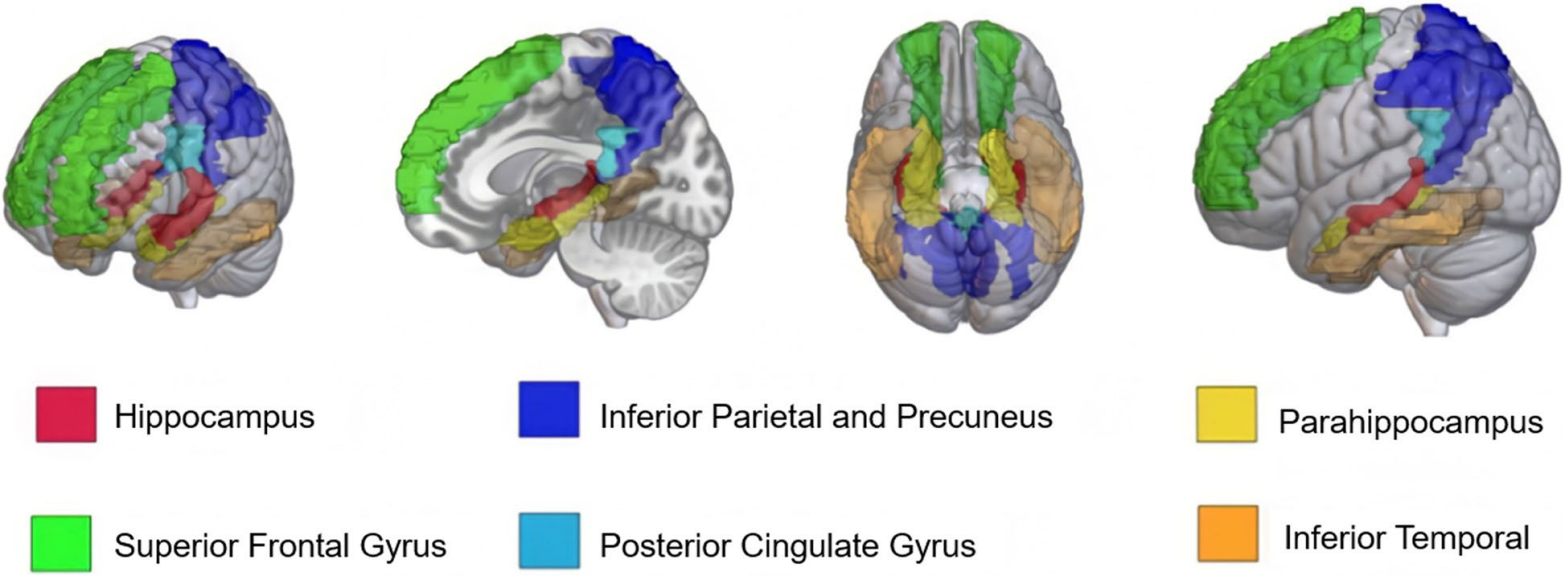
Representation of gray matter regions of interest (ROIs). Left to right: coronal, midsagittal, inferior axial, and lateral views of final gray matter ROIs. NDI and ODI were extracted from seven bilateral gray matter ROIs using the AAL-Atlas. The precuneus and inferior parietal lobe were combined into one ROI, “Precuneus + Inf. Parietal.” The six ROIs were then averaged bilaterally and included in logistic regression models. ROI figure was constructed in MRIcroGL using the AAL-atlas. Regions correspond to figure legend and are as follows: green = superior frontal gyrus, yellow = parahippocampus, red = hippocampus, orange = inferior temporal, dark blue = precuneus and inferior parietal, light blue = posterior cingulate gyrus

To develop robust norms representative of AD-neurodegeneration, and to then interrogate the diagnostic predictive utility of NODDI microstructure in AT(N) defined AD, while limiting multiple comparisons, we selected six bilateral gray matter and six white matter ROIs that have previously been identified as showing AD-related change on T1-weighted-MRI and DTI, respectively [7, 11, 15, 20, 37, 38]. Gray matter ROIs defined on the AAL atlas [39] and included the following regions: superior frontal gyrus, parahippocampus, hippocampus, inferior temporal lobe, posterior cingulate gyrus, and inferior parietal and precuneus, a combined ROI of bilateral inferior parietal lobe and precuneus (Fig. 1). White matter ROIs, defined by the JHU-tractography probability maps [40] as developed by Hua et al., included the following: cingulum (cingulate gyrus), cingulum (hippocampus), superior longitudinal fasciculus, inferior longitudinal fasciculus, uncinate fasciculus, and forceps major (Fig. 2).

To extract NODDI metrics from gray matter in native diffusion space, a T1-weighted atlas-space image was first nonlinearly warped into each subject’s native diffusion space. To improve the registration in this step especially in gray matter, we first constructed a ‘pseudo T1’ image in diffusion space [19, 21]. For this step, a gray matter (GM) fraction map was estimated by subtracting a white matter (WM) fraction map (estimated from a subject’s FA map using Atropos in ANTS) and a CSF fraction map (estimated from the Viso parameter map) from 1. The GM, WM, and CSF fraction maps were then multiplied by their respective tissue classes (CSF = 0, white matter = 1, gray matter = 2) and summed to create a pseudo T1 image. This pseudo T1 image has similar tissue contrast to a standard T1 image, which improves registration between diffusion and T1-weighted atlas space image. Using antsRegistration in ANTS, a nonlinear registration step was then performed to register the T1-weighted atlas-space template image to the pseudo T1 image for each subject. The resulting warp field was then used to warp AAL atlas-space ROIs to the subjects’ diffusion space. To ensure ROIs only included gray matter voxels, we used each subject’s gray matter fraction maps (thresholded at 0.7 and binarized) to filter each ROI. FSL’s *fslstats* was then used to extract mean values for NDI and ODI within each gray matter-masked masked ROI [21, 41].

To extract NODDI metrics from white matter, first, an *fsl* JHU atlas-space FA template image was nonlinearly registered to each subject’s native diffusion-space FA image. Next, the resulting warp fields were then applied to warp atlas-space JHU tractography probability maps to the subject’s native diffusion space. JHU tractography probability maps were generated as described in Hua et al. [35] for the regions of interest. Finally, these subject-space tract probability maps were then used to extract weighted average values for NDI and ODI within each tract metric (weighted by tract probability). Utilizing JHU tractography probability maps ensures that the average ROI value is more heavily weighted towards the core of the tract as opposed to the borders of the tract, where partial volume effects are likely greater.

### Statistical analysis

#### Robust normative analysis

To define “normal” neurodegeneration we generated internal normative distributions. The problem of developing normative distributions for metrics in a small disease population has been addressed previously in neuropsychological studies by developing internal norms for commonly used cognitive assessments [26–28]. In this study, we extended the robust norms approach from cognitive data to capture normal and abnormal ODI and NDI-based neurodegeneration in our sample as follows. First, we identified a “normal, presumed non-neurodegeneration” group as CU individuals who were negative on both CSF amyloid and tau (A − T −) (*n* = 231) and thus non-AD within both a clinical framework as well as within the AT(N) framework (2). Next, we ran regression models with age and sex in the model predicting each NODDI measure (see Supplement, Eq. 1) developing initial *z*-scores based on these predicted values (Eq. 2). Those who had a NODDI metric 1.5 or more standard deviations below expected for one or more ROIs were removed from the sample (*n* = 18) to ensure that the group was comprised of a robustly normal “non-neurodegeneration” subset. Using the reduced subset (*n* = 213) we ran the models again and saved the beta coefficients and root mean squared error (RMSE) for use in Eqs. 1 and 2, respectively, for each ODI and NDI metric and all CU, and MCI/ AD participants. From Eq. 1 we obtained predicted ODI and NDI values using the age and sex coefficients from the normative sample, and Eq. 2was used to convert predicted ODI or NDI values into a robust *z*-score.

1 $$\mathrm{Predicted}\;\mathrm{NDI}\;\mathrm{or}\;\mathrm{ODI}\;\mathrm{value}={\mathrm b}_0+b_{\mathrm{Age}\;}\times\;\mathrm{Age}\;(\mathrm{centered})+b_{\mathrm{Gender}\;}\times\;\mathrm{Gender}\;(0=\mathrm{male};\;1=\mathrm{female})$$

2 $$\mathrm{Robust}\;z-\mathrm{score}=(\mathrm{Observed}\;\mathrm{score}-\mathrm{Predicted}\;\mathrm{score})/\mathrm{RMSE}$$

We hypothesized that if NODDI metrics were indeed sensitive to the presence of neurodegeneration, the robust NDI and ODI *z*-scores would differ between the full CU group (*n* = 231) and the combined MCI/AD diagnosis group (*n* = 11). We further hypothesized that ODI and NDI *z*-scores would differ between CU A − (*n* = 231), CU A + /T − (*n* = 26), and CU A + /T + groups (*n* = 28), reflecting preclinical neurodegeneration. We tested these hypotheses using Kruskal–Wallis tests (FDR correction) followed with pairwise comparisons. We calculated Cliff’s delta effect sizes; these are less influenced by outliers and non-normative sample distributions than others such as Cohen’s *d* [42–44].

#### General logistic regression models

CSF biomarker status is considered the gold standard for the most sensitive detection of early pathologic AD. To investigate and compare whether diagnostic prediction of AD/MCI is improved by inclusion of NODDI metrics, we compared general logistic regression models that included CSF A/T status measures only, NODDI metrics for 6 ROIs only, and combined NODDI metric and CSF A/T status measures; we used penalized maximum likelihood estimation (Firth correction) to minimize bias in estimation of coefficients and associated confidence intervals [45, 46]. In part due to our small sample size, we limited in the number of predictor parameters in the analysis (i.e., number of ROIs).

We constructed the following models, each controlling for age and sex: (0) age and sex only (to evaluate how adding biomarker-related variables affected model performance); (1) CSF A/T status group (1 = A − T − , 2 = A + T − , 3 = A + T +), (2) WM NDI (3) WM ODI (4) GM NDI (5) GM ODI (6) CSF A/T status + WM NDI, (7) CSF A/T status + WM ODI (8) CSF A/T status + GM NDI, and (9) CSF A/T status + GM ODI. All 6 gray or white matter bilaterally averaged raw ROI values are included as individual covariates in the corresponding NODDI models ((Models 2–9). For ROIs, see Figs. 1 and 2; for models, see Table 2). Generalized Logistic Regression models with Firth bias correction were run using the *logistf* package (v1.24) in R studio (v1.1.463).

**Table 2:** Logistic regression model performance predicting CU and combined MCI/AD clinical status

Logistic regression models	AUC (95% CI)	AIC	PLR
All models including age and sex			
(0) No AT(N) predictors	(0) 0.74(0.60–0.88)	(0) − 27.1	(0) N/A
(1) CSF A/T status	(1) 0.93(0.89–0.97)	(1) 108.7	(1) − 102.68
(2) WM NDI	(2) 0.90(0.85–0.96)	(2) − 23.7	(2) 39.65
(3) WM ODI	(3) 0.81(0.65–1.00)	(3) − 5.7	(3) 21.61
(4) GM NDI	(4) 0.83(0.68–0.98)	(4) − 10.6	(4) 26.62
(5) GM ODI	(5) 0.87(0.78–1.00)	(5) 4.9	(5) 11.10
(6) CSF A/T status + WM NDI	(6) 0.96(0.93–0.99)	(6) 15.6	(6) 2.42
(7) CSF A/T status + WM ODI	(7) 0.97 (0.95–0.99)	(7) 25.7	(7) − 7.42
(8) CSF A/T status + GM NDI	(8) 0.97(0.95–0.99)	(8) 22.2	(8) − 4.20
(9) CSF A/T status + GM ODI	(9) 0.98(0.96–1.00)	(9) 27.6	(9) − 9.64

Logistic regression with Firth reduction predicted binomial clinical diagnosis outcomes (CU or MCI/AD). CU status included participants with CU diagnosis (*n* = 285) and A − /T − , A + /T − , or A + /T + CSF status. MCI/AD status included MCI or AD-diagnosed participants (*n* = 11) with A + /T − or A + /T + CSF A/T status. All models are controlled for age and sex. Receiver operator analysis (ROC) with area under the curve (AUC) assessed model prediction accuracy. Akaike information criteria (AIC) and penalized likelihood ratio (PLR) assessed model performance. Models with NODDI + CSF A/T status covariates had higher AUC values than the CSF A/T status-only model. The NODDI + CSF A/T status model with the lowest AIC and highest PLR included NODDI-NDI in AD-associated white matter regions

We then compared the diagnostic accuracy of the various models with receiver operator curve (ROC) area under the curve (AUC) analysis. We compared the diagnostic accuracy of the CSF A/T status-only model to that of models with both NODDI metrics and CSF A/T status, and to models with NODDI metrics alone. For each model, the ROC curve and AUC with a 95% confidence interval were generated using the *pROC* package (v1.16.1) in R studio.

Model fit was assessed with the Akaike information criteria (AIC) using the *extractAIC* function from the *stats* (v 3.6.2) R package, and also with the penalized log-likelihood ratio (PLR) for Firth reduction using the *anova.logistf function* in the *logistf (v 1.24)* package in R. Model fit was used to identify the best performing model when there were overlapping AUC 95% confidence intervals. Supplementary analyses were run dividing the impaired participants into separate MCI and AD groups (see Supplementary materials).

## Results

### Comparison of NODDI robust normative z-scores between clinical diagnosis groups and AT status groups

**Fig. 3:**
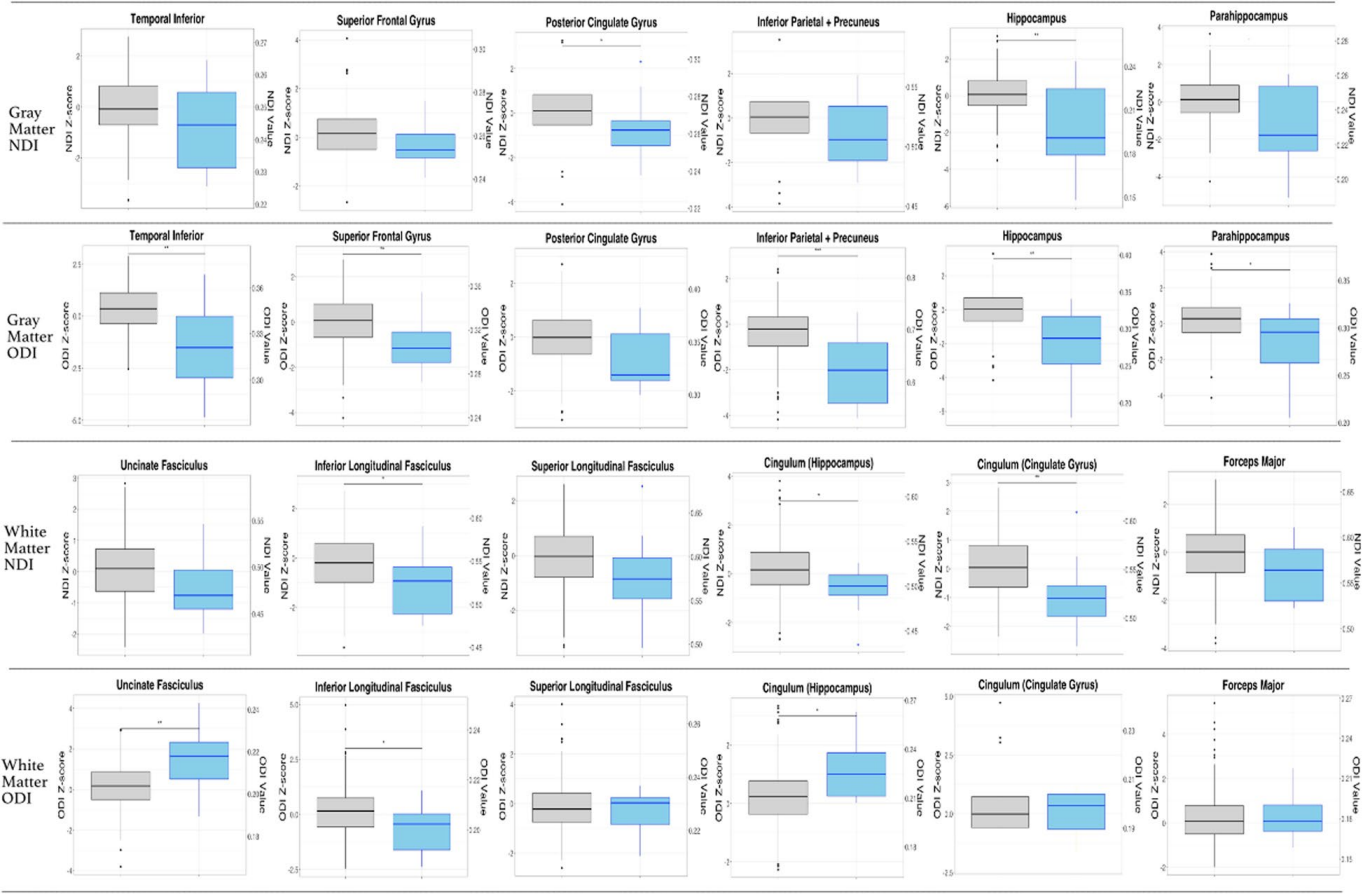
Distribution of NODDI metric *z*-scores across CU and MCI/AD clinical diagnosis groups for all ROIs. Boxplots displaying median, 25th quartile, and 75.^th^ quartile NDI or ODI *z*-scores and NDI or ODI values, respectively, for each ROI across CU and MCI/AD clinical status groups. NODDI-NDI and NODDI-ODI *z*-scores were developed using robust normative analysis in *R* as described. Box plot color corresponds to the clinical status group (gray = CU; blue = MCI/AD). ODI or NDI *z*-scores are labeled on the left *Y*-axis. On the right *Y*-axis are the raw NDI or ODI values corresponding to each z-score quartile. * indicates a significant difference in NDI *z*-score distribution between clinical diagnosis groups. “*” = *P* < 0.05, “**” = *P* < 0.01, “***” = *P* < 0.001, Kruskal–Wallis)

Comparison of NDI and ODI *z*-scores between cognitively unimpaired and impaired participants (CU vs. MCI/AD) showed significant differences in mean NDI or ODI *z*-score for all regions of interest except for the SLF and forceps major (Kruskal–Wallis, *p* < 0.05) (Table 3, Fig. 3). ODI and NDI *z*-scores correlated with p-Tau and amyloid across clinical status categories (Supplemental Figs. 1– 4). In gray matter regions, mean orientation dispersion (ODI) z-score was lower across all ROIs in cognitively impaired (MCI/AD) compared to unimpaired (CU) groups (Table 3). Mean NDI *Z*-score was significantly lower in the bilateral hippocampus, parahippocampus, and posterior cingulate gyrus for the MCI/AD group (Kruskal–Wallis, *p* < 0.05, Table 3, Fig. 3).

**Table 3:** Comparison of robust NODDI *z*-scores between CU and MCI/AD clinical status participants

GM ROIs (bilateral)	NDI ***z***-score ***p***-value (Kruskal–Wallis)	Cliff’s delta (95% CI)	ODI ***z***-score ***p***-value (Kruskal–Wallis)	Cliff’s delta (95% CI)
Superior frontal gyrus	0.11	0.28 (− 0.09–0.58)	0.002^b^	0.54 (0.17–0.78)^d^
Hippocampus	0.0061^b^	0.49 (0.002–0.79)^d^	0.001^b^	0.58 (0.13–0.83)^d^
Posterior cingulate gyrus	0.014^a^	0.44 (− 0.005–.73)	0.06	0.38 (− 0.018–0.67)
Inf. parietal + precuneus	0.15	0.25 (− 0.19–0.61)	0.00042^c^	0.63 (0.25–0.84)^d^
Parahippocampus	0.06	0.38 (− 0.13–0.73)	0.021^a^	0.41 (− 0.0150–0.71)
Temporal inferior	0.19	0.23 (− 0.22–0.60)	0.0026^b^	0.53 (0.06–0.81)^d^
WM ROIs (bilateral)				
Uncinate fasciculus	0.059	0.33 (− 0.02–0.61)	0.003^b^	− 0.53 (− 0.78 to − 0.13)^d^
Superior longitudinal fasciculus	0.08	0.31 (− 0.09–0.63)	0.614	0.09 (− 0.25–0.41)
Inferior longitudinal fasciculus	0.017^a^	0.43 (0.04–0.70)^d^	0.042^a^	0.36 (− 0.018–0.65)
Cingulum (hippocampus)	0.03^a^	0.44 (0.16–0.65)^d^	0.01^a^	− 0.45 (− 0.68 to − 0.14)^d^
Cingulum (cingulate gyrus)	0.005^a^	0.50 (1.10–0.76)^d^	0.74	0.06 (− 0.41–0.31)
Forceps major	0.068	0.32 (− 0.07–0.63	0.92	0.017 (− 0.35–0.322)

*Z*-scores for NODDI-ODI and NODDI-NDI were calculated with robust norms analysis as described. CU clinical diagnosis group included all participants with a clinical diagnosis of cognitively unimpaired (CU) (*n* = 285). MCI/AD clinical diagnosis group included all participants with a clinical diagnosis of either MCI (*n* = 6) or AD (*n* = 5). Effect size was calculated by Cliff’s delta with 95% confidence intervals

^a^ = *P* < 0.05

^b^ = *P* < 0.01

^C^ = *P* < 0.001, Kruskal–Wallis

^d^ = Sig. Cliff’s delta)

Within white matter regions, mean NDI *z*-score was lower in MCI/AD compared to CU in the inferior longitudinal fasciculus (ILF), cingulum (hippocampus), and cingulum (cingulate gyrus). Notably, ODI *z*-score showed significant bi-directionality within white matter, whereby bilateral UF and cingulum (hippocampus) were significantly higher in the MCI/AD group compared to the CU group, while the ILF was lower in the MCI/AD group compared to the CU group (Kruskal–Wallis, *p* < 0.05, Table 3).

**Fig. 4:**
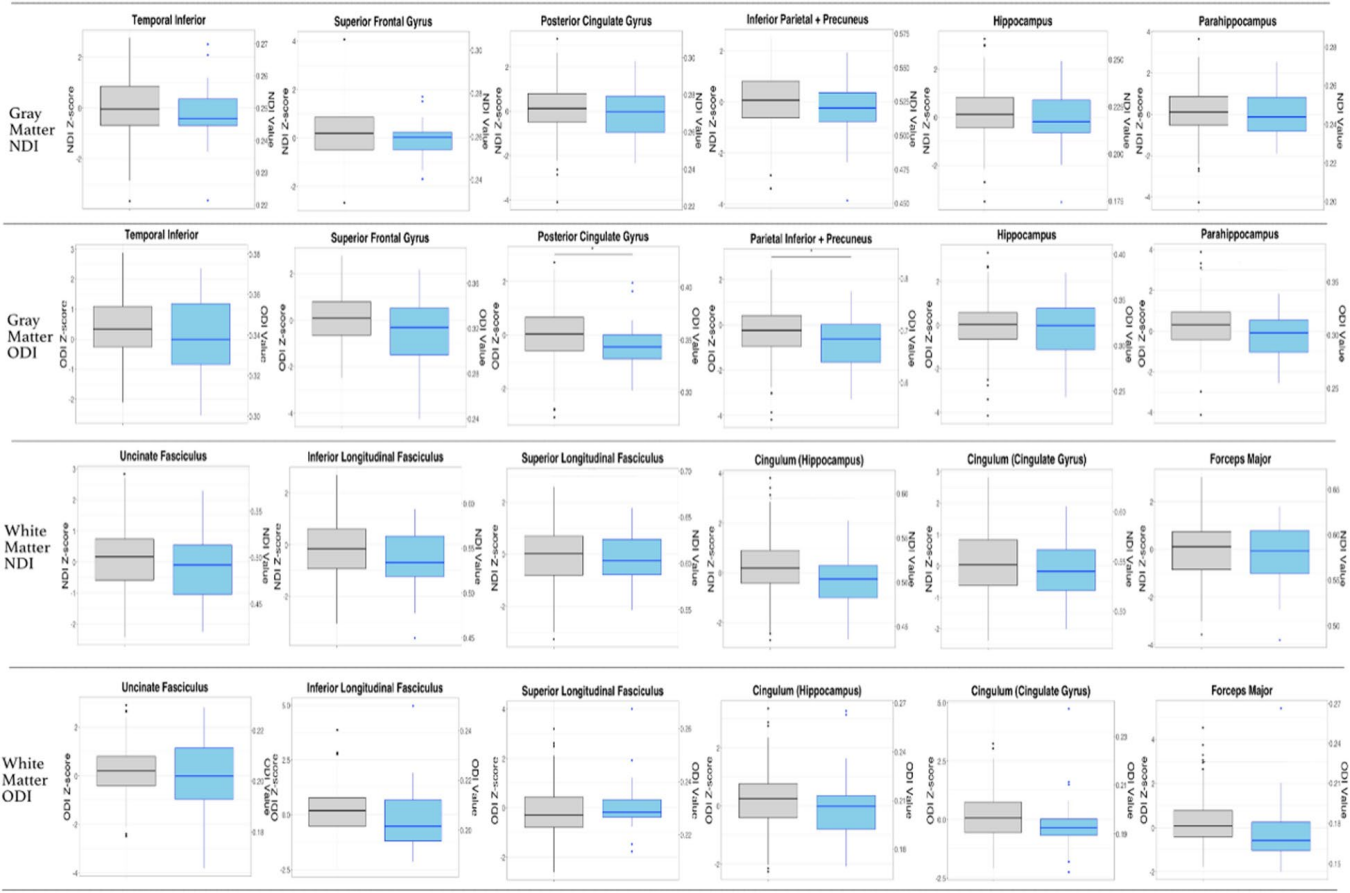
Distribution of NODDI metric *z*-scores across CU A − T − and A + T + CST AT status participants for all ROIs. For cognitively unimpaired participants, boxplots display the median, 25th quartile, and 75th quartile NDI or ODI *z*-scores and NDI or ODI values, respectively, for each ROI across A − T − or A + T + CSF A/T status groups. NODDI-NDI and NODDI-ODI *z*-scores were developed using robust normative analysis in *R* as described. CSF A/T status cutoffs were determined by ROC analysis as described. Box plot color corresponds to CSF A/T status (gray = A − T − ; blue = A + T +). ODI or NDI *z*-scores are labeled on the left *Y*-axis. On the right *Y*-axis are the raw NDI or ODI values corresponding to each z-score quartile. * indicates a significant difference in NDI *z*-score distribution between clinical diagnosis groups. “*” = *P* < 0.05, “**” = *P* < 0.01, “***” = *P* < 0.001, Kruskal–Wallis)

To evaluate whether microstructural changes were present based on A or T status, in the absence of differences in clinical status between groups, we compared NODDI metric *z*-scores between A − /T − , A + /T − , and A + /T + CSF status within CU individuals. We performed an omnibus test to identify ROIs that showed differences in *Z*-scores between A − /T − A + /T − A + /T + status. From ROIs that showed a difference in *Z*-scores, we performed follow-up pairwise comparisons across CSF A/T status groups. We did not observe a significant difference in NODDI metric *z*-scores between pairwise comparison of the A − /T − and A + /T − participants or the A + /T − , A + /T + participants. However, pairwise comparison between A − /T − and A + /T + individuals, differed significantly in mean ODI *z*-score in the posterior cingulate gyrus and combined inferior parietal and precuneus ROIs (Fig. 4, Table 4), whereby the A + T + group showed lower mean ODI *z*-scores. A + /T + participants trended towards a lower mean ODI z-score in the forceps major compared to the A − /T − group (Kruskal–Wallis, *p* = 0.055, Table 4, Fig. 4). No significant differences in mean NDI *z*-scores were observed for gray or white matter regions (Table 4).

**Table 4:** Comparison of mean robust NODDI *z*-scores between CU A − T − and A + T + CSF A/T status participants

GM ROIs (bilateral)	NDI *z*-score *p*-value (Kruskal–Wallis)	Cliff’s delta (95% CI)	ODI *z*-score *p*-value (Kruskal–Wallis)	Cliff’s delta (95% CI)
Superior frontal gyrus	0.30	0.17 (− 0.03–0.35)	0.06	0.22 (− 0.037–0.45)
Hippocampus	0.30	0.12 (− 0.13–0.35)	0.2	0.06 (− 0.19–0.31)
Posterior cingulate gyrus	0.30	0.16 (− 0.096–0.40)	0.01^a^	0.30 (0.09–0.49)^d^
Inf. parietal + precuneus	0.30	0.18 (− 0.04–0.39)	0.01^a^	0.23 (0.027–0.46)
Parahippocampus	0.40	0.12 (− 0.13–0.36)	0.06	0.24 (− 0.010–0.47)
Temporal inferior	0.40	0.11 (− 0.11–0.32)	0.06	0.16 (− 0.10–0.40)
WM ROIs (bilateral)				
Uncinate fasciculus	0.18	0.16 (− 0.08–0.38)	0.42	0.03 (− 0.24 to − 0.30)
Superior longitudinal fasciculus	0.61	0.04 (− 0.19–0.26)	0.68	− 0.11 (− 0.30–0.08)
Inferior longitudinal fasciculus	0.16	0.19 (− 0.05–0.41)	0.11	0.21 (− 0.05–0.44)
Cingulum (hippocampus)	0.09	0.24 (0.014–0.45)^d^	0.19	0.12 (− 0.12 to − 0.35)
Cingulum (cingulate gyrus)	0.16	0.14 (− 0.07–0.35)	0.29	0.21 (0.01–0.40)^d^
Forceps major	0.62	0.06 (− 0.18–0.29	0.055	0.38 (0.16–0.60)^d^

*Z*-scores for NODDI-ODI and NODDI-NDI were calculated with robust norms analysis as described. CSF A − T − status group included participants with a clinical diagnosis of cognitively unimpaired (CU) and A − T − CSF (*n* = 231). A + T + CSF (*n* = 28) status group included CU-diagnosed participants with A + T + CSF. CSF status cutoffs were determined by cutoffs through ROC analysis as described. Omnibus comparison (KW test, FDR correction) evaluated mean difference in ROIs *z*-score across all CSF groups. For ROIs that had a significant difference in *Z*-score across groups with omnibus comparison, an additional follow-up pairwise comparison (KW) was conducted across CSF status groups (A − /T − , A + /T +). All ROIs that showed significance in the omnibus test showed significance difference in pairwise comparison test. Thereby in this table, ROIs with non-significant *P*-values were those that only underwent initial omnibus comparison, whereas ROIs with significant *p*-values underwent both omnibus and pair-wise comparison testing. Effect size was calculated by Cliff’s delta with 95% confidence intervals

^a^ = *P* < 0.05

^b^ = *P* < 0.01

^c^ = *P* < 0.001, Kruskal–Wallis

^d^ = Sig. Cliff’s delta)

### General logistic regression comparing diagnostic accuracy of NODDI metrics and CSF A/T status

Table 2 summarizes results across all models predicting clinical diagnosis (MCI/AD vs CU). All models including at least one AT(N) predictor showed higher AUC and nominally improved model fit compared to model 0; however, models with CSF A/T status outperformed the base model (i.e., AUC CI’s higher than model 0). For models that included both NODDI metrics and CSF A/T status (Models 6–9) as predictors of clinical status, AUC values and 95% confidence intervals are closer to 1.00, than models that included CSF A/T status only (Model 1) (Table 2). Participant A/T biomarker classification was unchanged regardless of whether p-Tau Aβ42 cut-offs or Aβ42/Aβ40 and p-Tau cut-offs were used.

Exploratory analysis where MCI and AD groups were not combined, demonstrated that models which included both the NODDI metrics and CSF A/T status together, also had higher comparative AUC values than the CSF A/T status model alone, when predicting CU vs. MCI and CU vs. AD groups (Supplemental Tables 1 and 2).

When predicting CU vs. MCI/AD diagnosis, the model with the best fit (lowest AIC and most positive PLR) included WM NDI and CSF A/T status (Table 2). Likewise, when predicting CU vs. AD, the model with the lowest AIC and most positive PLR included both NODDI WM NDI + CSF A/T status (Supplemental Table 1). For predicting CU vs. MCI clinical status, both the NODDI WM ODI + CSF A/T status and NODDI WM NDI + CSF A/T status model had similar AIC and PLR values, differing by 0.2, respectively (Supplemental Table 2).

## Discussion

While AD is biologically characterized by amyloid plaques (A) and neurofibrillary tangles (T), these features do not always correlate well with clinical cognitive decline [2, 3], and other disease features are needed to fully inform on clinical progression. Features of neurodegeneration (N) have long been studied in the context of AD and indeed in vivo methods such as structural imaging long precede the development of neuroimaging techniques that are sensitive to pathologic amyloid and tau. Still, the efforts to define normal vs. abnormal neurodegeneration using neuroimaging among healthy and AD continuum individuals have been limited, not for lack of imaging methods, but rather due to limitations in separating age-related from disease-related effects. While several studies have compared markers of neurodegeneration between different clinical categories of AD (AD vs. CU or MCI vs. CU) [20, 21, 37, 47], “normal” (AD neurodegeneration absent) and “abnormal” neurodegeneration (AD neurodegeneration present) remains largely undefined among cognitively unimpaired individuals. As participants in this study were characterized on CSF A/T status, it was possible to examine neurodegeneration within preclinical, A/T criteria defined AD.

By and large, our findings support our hypotheses that (1) mean robust z-scores of NDI and ODI would be lower for cognitively impaired (MCI/AD) individuals compared to CU participants reflecting greater neurodegeneration, (2) that among cognitively unimpaired participants, individuals with greater A/T pathology would show greater neurodegeneration based on NDI and ODI *z*-scores compared to individuals who were negative for AD pathology (A − /T −), and (3) that adding NODDI metrics to logistic regression models would improve prediction of clinical status and model performance. Overall, robust *z*-scores differing between biologically and clinically defined groups suggest greater neurodegeneration with greater disease severity. The exception to results largely supporting our expectations was the orientation dispersion findings in UF and cingulum (hippocampus) white matter regions, which showed bidirectional differences in mean ODI *z*-score between CU and MCI/AD groups.

Measures of neurodegeneration, while commonly studied in the context of AD are typically considered to be non-specific markers of dementia [2]. Here, we capitalized on prior work to select gray and white matter regions of interest previously shown to be affected by AD to determine the extent to which microstructural alterations in these regions could be separated into normal vs. abnormal neurodegeneration, as well as inform upon preclinical neurodegeneration in the context of known A/T status. Gray matter regions were those known to be sensitive to amyloid and tau pathology, [48–53] while the white matter regions included were those that connect cortical regions subserving memory function [54, 55] and long association fibers [56, 57] previously observed to be vulnerable to AD.

Counter to our expectations of observing decreased ODI among individuals with MCI and AD, we observed bidirectional effects when comparing ODI between cognitively unimpaired and impaired groups in several white matter regions, depending on the brain region examined. While this is unexpected, it is possible that regional reactive astrogliosis that is observed as clinical disease progresses to cognitive impairment could impact ODI measurements. While reactive astrogliosis in the white matter has been largely related to Aβ plaque deposition, studies focusing on tauopathies and AD-associated tau deposition, have shown that microglia and astrocytes also become active following tau accumulation and NFT formation [52, 58–60]. Similar to our findings, a cMD microstructural imaging study by Montal et al., showed a biphasic relationship between changes in gray matter cMD and clinical cognitive decline among A/T biomarker-positive individuals, which they proposed to be related to reactive astrogliosis [15]. High NODDI-ODI values have also been observed in a rodent tau pathology model of AD [61]. Additionally, higher values of other diffusion imaging metrics have been associated with reactive astrogliosis. Zhou et al. (2011) found that increased mean diffusion kurtosis was associated with higher astrogliosis as measured by immunohistochemistry in traumatic brain injury [62]. Another possibility for increased ODI in regions such as UF and ILF is that loss of fibers in a largely parallel-running long-projecting tract could effectively increase orientation dispersion [36]. Additional studies will be needed to replicate these findings, as well as longitudinal studies to determine how regional ODI changes over time during disease progression.

Using robust NDI and ODI *z*-scores allowed us to compare NODDI differences across A/T, preclinical, and clinical statuses. Even among cognitively unimpaired individuals, we observed differences in ODI *z*-scores among those who were positive for AD (A + /T +) compared to AD negative controls (A − /T −), in the precuneus, inferior parietal, and PCC gray matter regions. These findings suggest that detectable changes in microstructure, as indexed by orientation dispersion, may occur following or concurrent with A/T positivity, yet prior to development of clinical levels of impairment. Given that significant differences in ODI were observed between cognitively unimpaired A − /T − and A + /T + status participants, but not between cognitively unimpaired A − /T − and A + /T − individuals, the preclinical neurodegeneration observed here may reflect micro-architectural loss driven primarily by hyperphosphorylated-tau, that has a synergistic effect with amyloid [51, 52, 61, 63, 64]. However, because we did not observe significant differences in ODI between cognitively unimpaired A + /T − and A + /T + individuals, it is not entirely clear how to interpret the disparities in ODI between the A − /T − and A + /T + cohorts strictly in terms of plaque and tangle pathology.

Lower orientation dispersion in the precuneus, inferior parietal, and posterior cingulate regions among CU participants likely reflects loss of neuron and axon dispersion, or microstructural neural connectivity, in the highly dispersed gray matter regions, where hyperphosphorylation of tau results in the formation of neurofibrillary tangles [52]. This would be expected within the AT(N) framework of AD. One study by Rodriguez-Vieitez et al. found that changes in cMD in AD-associated areas were observed following tau, but not amyloid, deposition in CU individuals [63]. Further support comes from animal studies, where one study found that high NODDI-ODI values were sensitive to tau burden in a rodent tau pathology model of AD [61]. Findings from Vogt et al. suggest that neurodegenerative changes in gray matter may precede white matter degeneration [25, 41]. Likewise, the results of this study suggest that neurodegeneration in gray matter precedes that in white matter in the AD pathologic process; and, that gray matter neurodegeneration follows amyloid and tau and deposition. The observed decrease in ODI may reflect loss of complexity within highly dispersed cortical gray matter preceding loss of neuron and axon density. Given that our findings of decreased ODI were isolated to the comparisons between A + /T + and A − /T − CU participants, could be indicative of tau deposition having a synergistic effect with amyloid. However, we cannot rule out that these patterns were due to underpowered analyses, and replication studies are needed. Somewhat unexpectedly, no significant differences in NDI were found between cognitively unimpaired CSF A/T status groups in any brain region. This may suggest that substantial reductions in neurite density are indicative of more advanced stages of AD pathophysiology, and correspondingly, that extensive neurite loss is not a prominent feature of preclinical AD.

Our results from our logistic regression model comparison and receiver operator curve analyses showed that including NODDI metrics along with CSF A/T status improved the performance of logistic regression models when predicting cognitively impaired clinical status (MDI/AD) compared to models that relied solely on CSF A/T status alone. Participant A/T biomarker classification was unchanged regardless of whether p-Tau Aβ42 cut-offs or Aβ42/Aβ40 and p-Tau cut-offs were used. These results suggest that NODDI neuroimaging metrics add clinically useful information to disease prediction above and beyond A and T status.

Altered microstructure that is linked to AD processes among cognitively unimpaired participants has been observed in other studies. In a comparison of AD polygenic risk scores (PRS), Foley et al. [65] found altered cingulum microstructure among individuals with a higher PRS. In a study of autosomal dominant AD, Caballero et al. found that mutation carriers had altered diffusivity in the forceps major, forceps minor, as well as inferior and superior longitudinal fasciculi prior to the development of dementia [66–68]. A microstructural imaging study by Montal et al. that examined cMD in gray matter, found that cMD was increased for A + , but decreased among A + T + , in cognitively unimpaired participants [15]. Additionally, a previous study from our center has shown an interaction between CSF amyloid and tau on gray matter neurite density, but not cortical thickness, in cognitively unimpaired individuals [25], suggesting the possibility of a protracted neurodegenerative process in AD that spans several years, but that may have been underappreciated prior to the application of diffusion-weighted imaging. cMD has also been shown to have a synergistic interaction with A + T + burden, as assessed by PET imaging, on a cognitive slope [63]. These findings concur with our results suggesting that preclinical microstructural neurodegeneration follows amyloid and tau deposition and that diffusion-weighted imaging metrics may show promise for characterizing N in the AT(N) framework when used in conjunction with markers of amyloid and tau.

## Limitations

While promising, these results are cross-sectional, and longitudinal analyses are expected to better define the spatial patterns of neurodegenerative change over the course of AD progression. We capitalized on the opportunity to examine preclinical disease in a population of participants characterized on A and T status, comparisons by clinical status were limited by the few participants with impaired clinical disease statuses. The lack of amyloid or tau PET imaging in our cohort is a limitation, as the spatial/topographical relationship between molecular pathology and neurodegeneration was not assessed here. Furthermore, replication in additional cohorts is needed. Additionally, the CSF A + /T + cognitively unimpaired group size was small (*n* = 28). Given these methodological weaknesses, our findings with regards to improving the diagnostic prediction accuracy of AD need to be interpreted with caution and should be viewed as primarily exploratory in nature.

## Conclusion

In conclusion, we address a notable gap in AD research—defining “normal” and “abnormal” neurodegeneration in a preclinical population—by utilizing robust norm *z*-score analysis with metrics of NODDI ODI and NDI. Our findings suggest NODDI—and likely other multi-compartment models of diffusion—hold promise for detecting preclinical neurodegeneration. We show for the first time that NODDI NDI and ODI capture early, neurodegenerative AD pathology processes. We also provide evidence that including metrics of tissue microstructure indicative of neurodegeneration with CSF A/T status is more informative for predicting AD-clinical status than relying on CSF A/T status alone. This suggests that the neurodegeneration (N) captured by microstructural imaging, such as NODDI, may increase the clinical sensitivity of CSF A and T biomarkers. However, due to small sample sizes, we reiterate that our findings need to be interpreted with caution and should be viewed as exploratory in nature. While further work is needed to define N within the AT(N) framework, we demonstrate that diffusion-weighted imaging has potential as a future neuroimaging biomarker for the detection of preclinical neurodegenerative changes in AD.

### Supplementary Information

**Additional file 1:** Supplementary Materials and Methods: General Logistic Regression Models Analysis. General Logistic Regression Model Results: Supplementary Tables. **Supplementary Table 1.** Logistic Regression Model Performance Predicting CU and AD-Dementia Clinical Status. **Supplementary Table 2.** Logistic Regression Model Performance Predicting CU and MCI Clinical Status. **Supplemental Figure 1.** Correlation between NODDI ODI Z-score and CSF p-Tau Levels across ROIs. **Supplemental Figure 2.** Correlation between NODDI NDI Z-score and CSF p-Tau Levels across ROIs. **Supplemental Figure 3.** Correlation between NODDI ODI Z-score and CSF Aβ42/40 Levels across ROIs. **Supplemental Figure 4.** Correlation between NODDI NDI Z-score and CSF Aβ42/40 Levels across ROIs.

## Data Availability

Data used in this study are available upon reasonable request. Requests for resources can be made by accessing https://www.adrc.wisc.edu/apply-resources.
