# Supplementary material for: Neuroimaging of tissue microstructure as a marker of neurodegeneration in the AT(N) framework: defining abnormal neurodegeneration and improving prediction of clinical status
Source: Alzheimers Res Ther. 2023 Oct 17;15:180. doi: 10.1186/s13195-023-01281-y (PMC10583332; doi:10.1186/s13195-023-01281-y)
Supplement: Supplementary file 1 — Additional file 1: Supplementary Materials and Methods: General Logistic Regression Models Analysis. General Logistic Regression Model Results: Supplementary Tables. Supplementary Table 1. Logistic Regression Model Performance Predicting CU and AD-Dementia Clinical Status. Supplementary Table 2. Logistic Regression Model Performance Predicting CU and MCI Clinical Status. Supplemental Figure 1. Correlation between NODDI ODI Z-score and CSF p-Tau Levels across ROIs. Supplemental Figure 2. Correlation between NODDI NDI Z-score and CSF p-Tau Levels across ROIs. Supplemental Figure 3. Correlation between NODDI ODI Z-score and CSF Aβ42/40 Levels across ROIs. Supplemental Figure 4. Correlation between NODDI NDI Z-score and CSF Aβ42/40 Levels across ROIs. [file 13195_2023_1281_MOESM1_ESM.docx]

**Supplemental Information:**

**Supplementary Materials and Methods:**

**General Logistic Regression Models Analysis:**

Identical to our main methods, we constructed the following general logistic regression with Firth correction each controlling for age and sex: (0) age and sex only; (1) CSF A/T status group (1 = A-T-, 2 = A+T-, 3 = A+T+), (2) WM NDI (3) WM ODI (4) GM NDI (5) GM ODI (6) CSF A/T Status + WM NDI, (7) CSF A/T Status + WM ODI (8) CSF A/T Status + GM NDI, and (9) CSF A/T Status + GM ODI. The 6 bilaterally averaged gray and white matter ROIs previously described (Fig. 1, 2) were included in the respective corresponding models. Participants were separated into 3 groups based on clinical diagnosis status: CU (n = 281), MCI (N = 6), and AD (n=5). Models predicted outcome in a binomial fashion (CU vs. MCI and CU vs. AD). Models were generated in R Package *logistf (v 1.24).* Model fit was assessed with the Akaike Information Criteria (AIC) using the *extractAIC* function from the *stats* (v 3.6.2) R package, and also with the Penalized log likelihood ratio (PLR) for firth reduction using the *anova.logistf function* in the *logistf (v 1.24)* package in R. We compared the diagnostic accuracy of the various models with Receiver Operator Curve (ROC) Area Under the Curve (AUC) analysis generated using the *pROC* package (v1.16.1) in R studio.

**General Logistic Regression Model Results:**

Supplementary analyses show that models including NODDI metrics and CSF A/T status had the higher comparative AUC values than the CSF A/T Status model when predicting CU vs. MCI and CU vs. AD clinical outcomes (Suppl. Tables 1, 2).

**Supplementary Tables**

**Supplementary Table 1: Logistic Regression Model Performance Predicting CU and AD-Dementia Clinical Status**

| Logistic Regression Models | AUC (95% CI) | AIC | PLR |
| --- | --- | --- | --- |
| All models including age and sex  (0) No AT(N) Predictors  (1) CSF A/T Status  (2) WM NDI  (3) WM ODI  (4) GM NDI  (5) GM ODI  (6) CSF A/T Status + WM NDI  (7) CSF A/T Status + WM ODI  (8) CSF A/T Status + GM NDI  (9) CSF A/T Status + GM ODI | (0) 0.79(0.64-0.94)  (1) 0.95(0.89-1.00)  (2) 0.98(0.96-1.00)  (3) 1.00(1.00-1.00)  (4) 0.97(0.92-1.00)  (5) 1.00(0.99-1.00)  (6) 0.98(0.94-1.00)  (7) 1.00(0.99-1.00)  (8) 0.99(0.97-1.00)  (9) 0.99(0.98-1.00) | (0) -1.1  (1) 79.8  (2) 27.9  (3) 54.4  (4) 34.7  (5) 49.7  (6) 44.5  (7) 64.8  (8) 49.4  (9) 59.0 | (0) N/A  (1) -73.76  (2) -11.89  (3) -38.42  (4) -18.71  (5) -33.65  (6) -26.53  (7) -46.79  (8) -31.43  (9) -41.04 |

**Supplementary Table 1: Logistic Regression Model Performance Predicting CU and AD-Dementia Clinical Status**. Logistic regression with Firth Reduction predicted binomial clinical diagnosis outcomes (CU or AD). CU status included participants with CU diagnosis (n = 285) and A-/T-, A+/T-, or A+/T+ CSF status. AD status included AD diagnosed participants (n = 5) with A+/T- or A+/T+ CSF A/T status. All models controlled for age and sex. Receiver Operator Analysis (ROC) with Area Under the Curve (AUC) assessed model prediction accuracy. Akaike Information Criteria (AIC) and Penalized Likelihood Ratio (PLR) assessed model performance. Models with NODDI + CSF A/T Status covariates had higher AUC values than the CSF A/T Status only model. The NODDI + CSF A/T Status model with the lowest AIC and highest PLR included NODDI-NDI in AD-associated white matter regions.

**Supplementary Table 2: Logistic Regression Model Performance Predicting CU and MCI Clinical Status**

| Logistic Regression Models | AUC (95% CI) | AIC | PLR |
| --- | --- | --- | --- |
| All models including age and sex  (0) No AT(N) Predictors  (1) CSF A/T Status  (2) WM NDI  (3) WM ODI  (4) GM NDI  (5) GM ODI  (6) CSF A/T Status + WM NDI  (7) CSF A/T Status + WM ODI  (8) CSF A/T Status + GM NDI  (9) CSF A/T Status + GM ODI | (0) 0.75(0.55-0.95)  (1) 0.94(0.88-1.00)  (2) 0.92(0.86-0.99)  (3) 0.85(0.70-1.00)  (4) 0.86(0.72-1.00)  (5) 0.86(0.73-0.99)  (6) 0.97(0.94-1.00)  (7) 0.96(0.92-1.00)  (8) 0.97(0.95-1.00)  (9) 0.97(0.95-1.00) | (0) 1.30  (1) 102.2  (2) 24.2  (3) 19.6  (4) 28.3  (5) 27.7  (6) 42.2  (7) 42.7  (8) 49.4  (9) 48.3 | (0) N/A  (1) -96.25  (2) -8.28  (3) -3.59  (4) -12.34  (5) -11.69  (6) -24.23  (7) -24.64  (8) -31.43  (9) -30.35 |

**Supplementary Table 2: Logistic Regression Model Performance Predicting CU and MCI Clinical Status**. Logistic regression with Firth Reduction predicted binomial clinical diagnosis outcomes (CU or MCI). CU status included participants with CU diagnosis (n = 285) and A-/T-, A+/T-, or A+/T+ CSF status. MCI status included MCI diagnosed participants (n = 6) with A+/T- or A+/T+ CSF A/T status. All models controlled for age and sex. Receiver Operator Analysis (ROC) with Area Under the Curve (AUC) assessed model prediction accuracy. Akaike Information Criteria (AIC) and Penalized Likelihood Ratio (PLR) assessed model performance. Models with NODDI + CSF A/T Status covariates had higher AUC values than the CSF A/T Status only model. The NODDI + CSF A/T Status model with the lowest AIC and highest PLR included NODDI-ODI in AD-associated white matter regions.

**Supplemental Figure 1: Correlation between NODDI ODI Z-score and CSF p-Tau Levels across ROIs**.


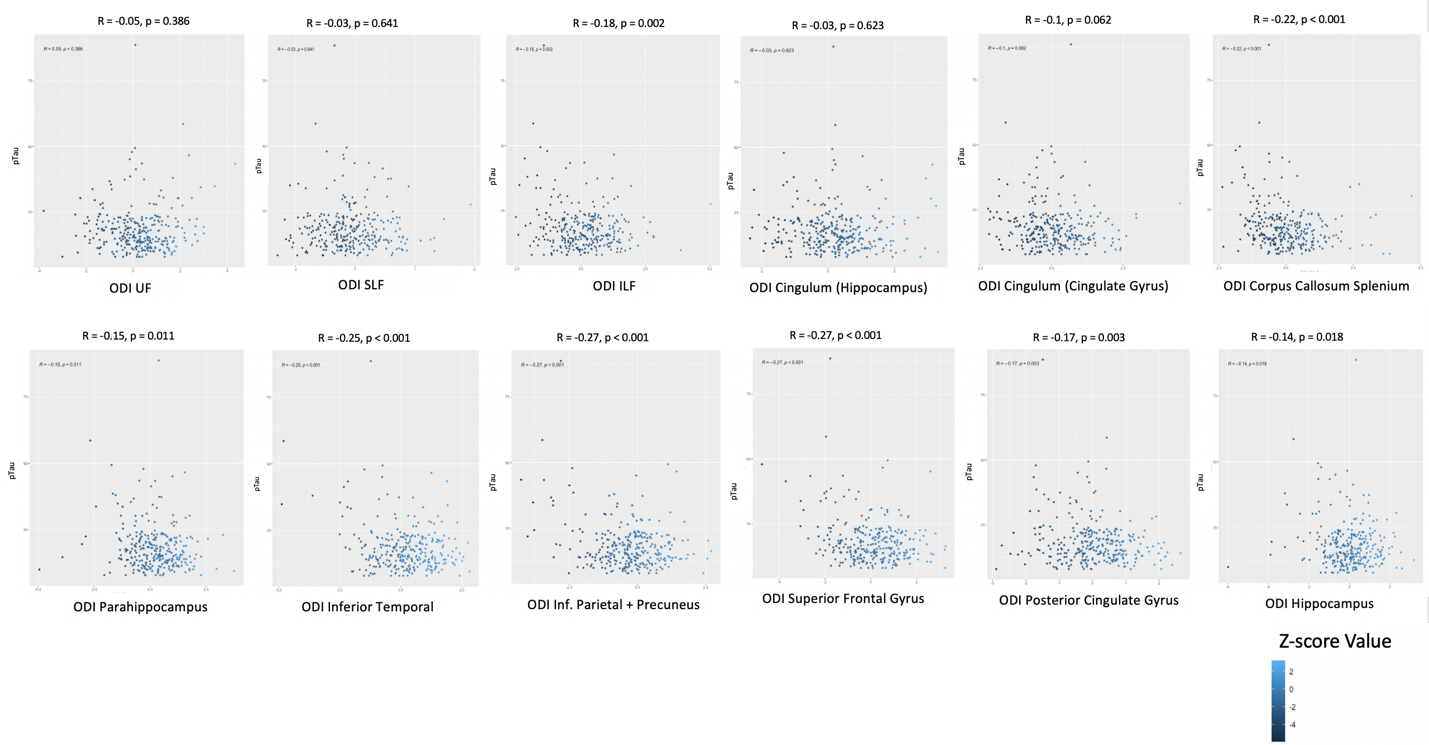


Top row: white matter regions. Bottom row: gray matter regions. Y-axis: CSF p-Tau levels (mg/ml). X-axis: ODI Z-scores for ROI. Blue hue corresponds to Z-score value going from negative to positive. ODI Z-scores had significant negative correlation with p-Tau level across all gray matter ROIs, and the ILF and corpus callosum (Splenium) white matter regions. ODI had a positive, non-significant correlation with p-Tau in UF.

**Supplemental Figure 2: Correlation between NODDI NDI Z-score and CSF p-Tau Levels across ROIs.**
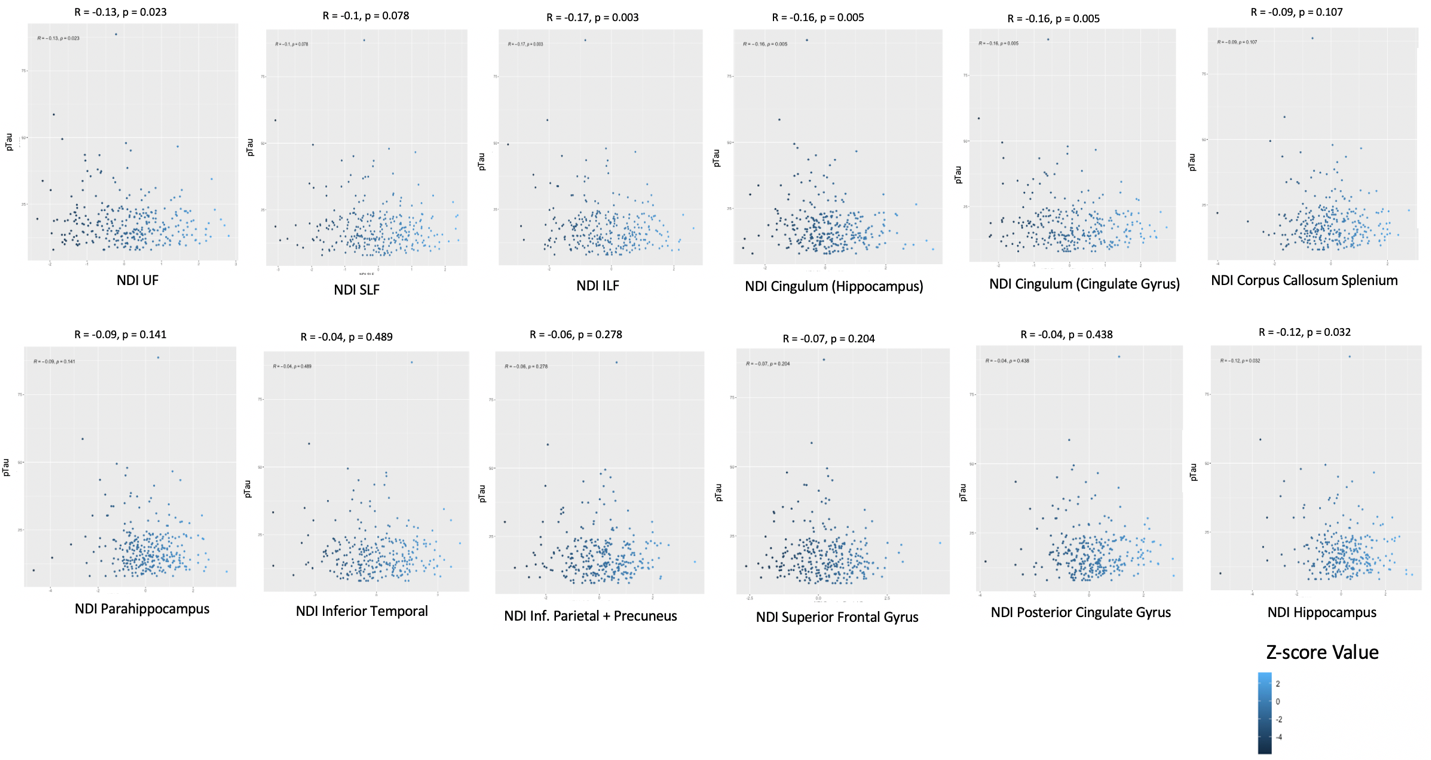
 Top row: white matter regions. Bottom row: gray matter regions. Y-axis: CSF p-Tau levels (mg/ml). X-axis: NDI Z-scores for ROI. Blue hue corresponds to Z-score value going from negative to positive. ODI Z-scores had significant negative correlation with p-Tau level for the Hippocampus, UF, Cingulum (Hippocampus), Cingulum (Cingulate Gyrus) ROIs.

**Supplemental Figure 3: Correlation between NODDI ODI Z-score and CSF Aβ42/40 Levels across ROIs.**


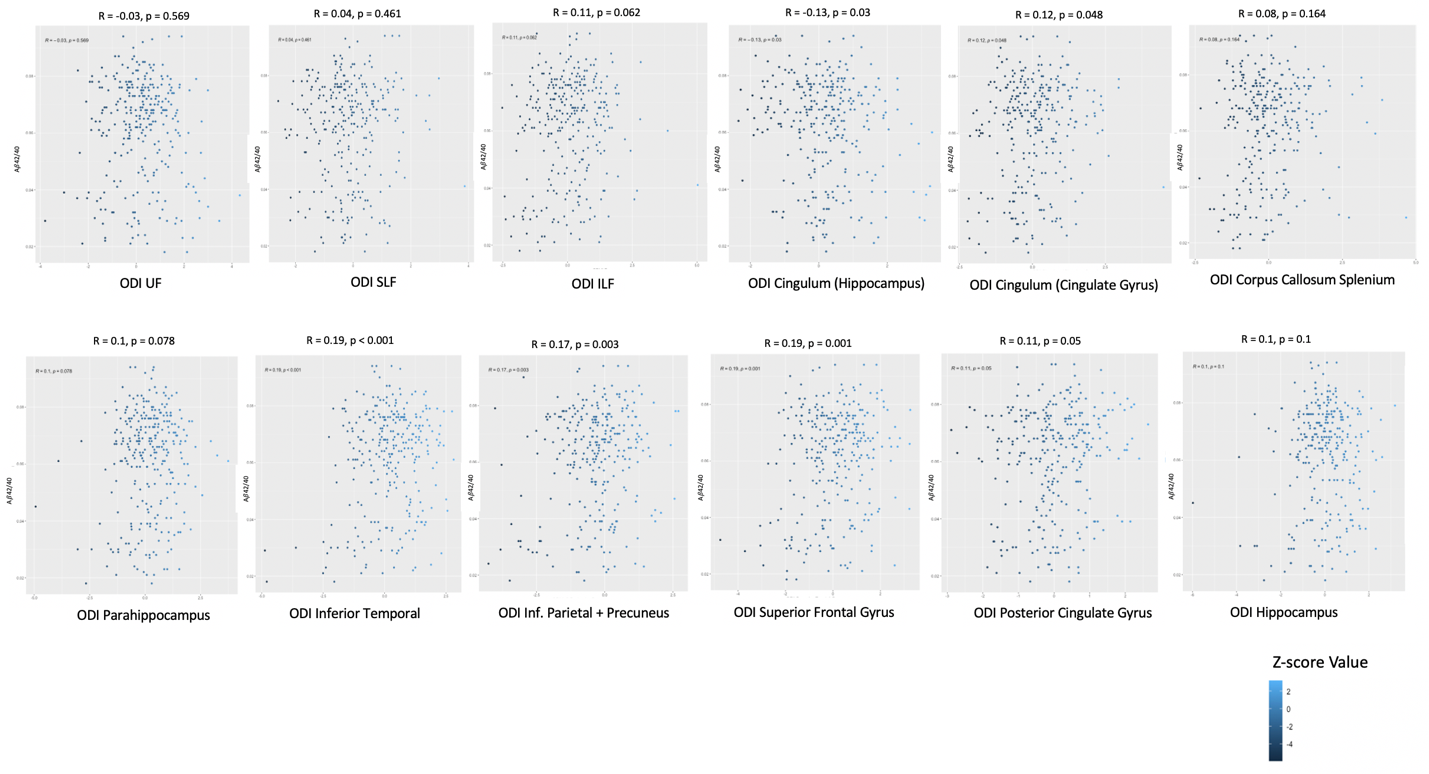
 Top row: white matter regions. Bottom row: gray matter regions. Y-axis: CSF **Aβ42/40** (mg/ml). X-axis: ODI Z-scores for ROI. Blue hue corresponds to Z-score value going from negative to positive. Significant positive correlations were found in Cingulum (Cingulate Gyrus) Posterior Cingulate Gyrus, Inferior Parietal + Precuneus, Inferior Temporal, Superior Frontal Gyrus. Cingulum (Hippocampus) showed a significant negative correlation between ODI and Aβ42/40.

**Supplemental Figure 4: Correlation between NODDI NDI Z-score and CSF Aβ42/40 Levels across ROIs**.


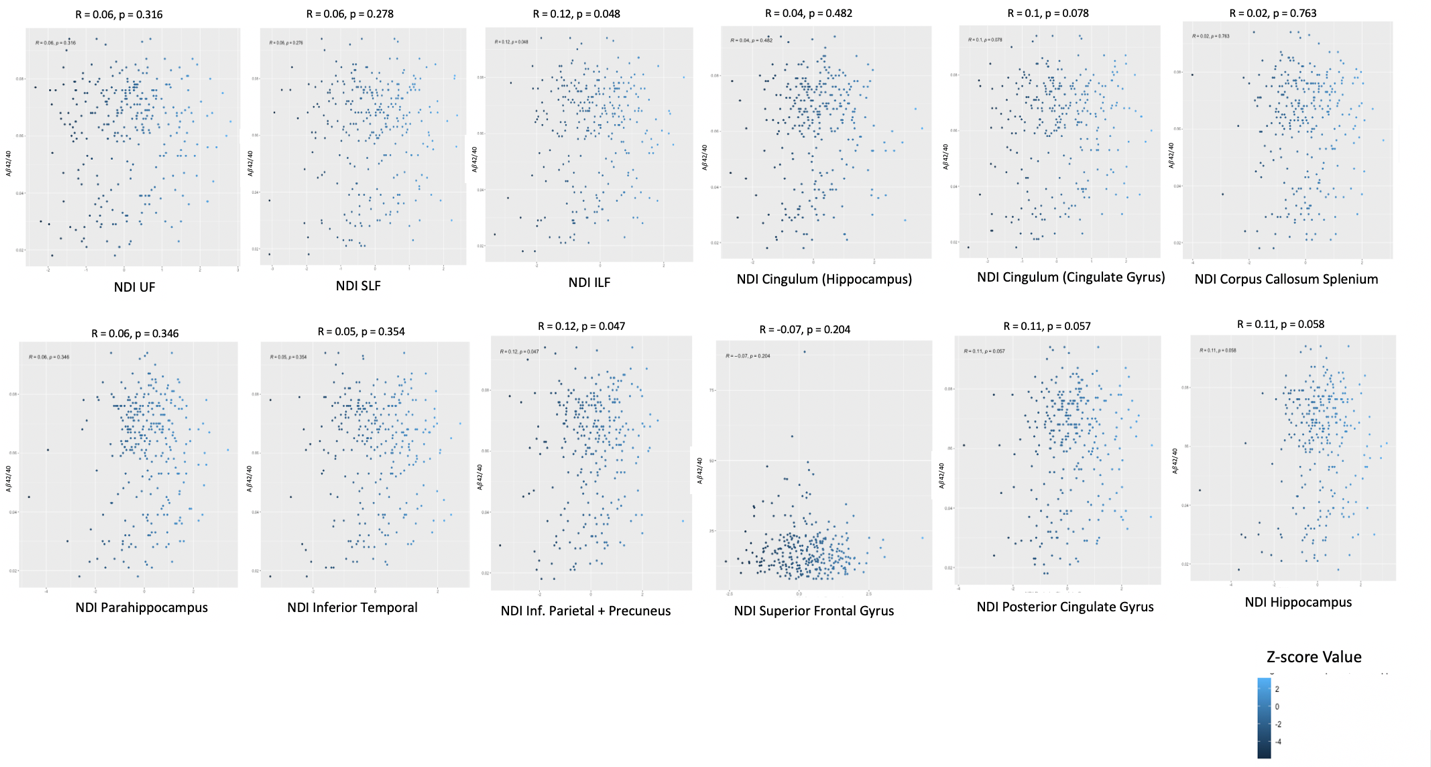


Top row: white matter regions. Bottom row: gray matter regions. Y-axis: CSF Aβ42/40 (mg/ml). X-axis: NDI Z-scores for ROI. Blue hue corresponds to Z-score value going from negative to positive. A significant positive correlation was found in the Inferior Parietal + Precuneus ROI.
